# Granulocyte-Colony Stimulating Factor-Induced Vasculitis Successfully Treated With Short-Term Corticosteroid Therapy: A Case Report

**DOI:** 10.7759/cureus.20563

**Published:** 2021-12-21

**Authors:** Shintaro Yamamoto, Daisuke Waki, Takeshi Maeda

**Affiliations:** 1 Department of Endocrinology and Rheumatology, Kurashiki Central Hospital, Kurashiki, JPN; 2 Department of Hematology and Oncology, Kurashiki Central Hospital, Kurashiki, JPN

**Keywords:** filgrastim, corticosteroid, carotidynia, aortitis, vasculitis, granulocyte-colony stimulating factor

## Abstract

Granulocyte-colony stimulating factor (G-CSF) is widely used for preventing neutropenia, and large vessel vasculitis has been recognized as one of its severe adverse events. We report a case of diffuse large B-cell lymphoma in a 78-year-old woman in whom fever and right cervical pain developed after administration of filgrastim. Computed tomography and cervical artery ultrasound imaging revealed wall thickening in the right common carotid artery. We diagnosed her with G-CSF-induced vasculitis and administered prednisolone of 50 mg/day (1 mg/kg/day) to her. Her symptoms disappeared in a few days, and prednisolone was discontinued six weeks after initiation. G-CSF-induced vasculitis may be improved with short-term high-dose corticosteroids with rapid tapering.

## Introduction

Granulocyte-colony stimulating factor (G-CSF) is widely used to prevent febrile neutropenia in patients undergoing chemotherapy. It is generally well-tolerated, except for splenic rupture, arterial thrombosis, and pulmonary toxicity [1]. Recently, large vessel vasculitis has been increasingly reported as one of its severe adverse events [2]. However, an optimal treatment strategy for G-CSF-induced vasculitis is still unclear. Herein, we report a case of large vessel vasculitis in the carotid artery occurring after administration of G-CSF that was successfully treated with short-term corticosteroid therapy.

## Case presentation

A 78-year-old Japanese woman was referred to our hospital for treatment of diffuse large B-cell lymphoma with a left lower leg mass. She was started on R-THP-COP (rituximab, pirarubicin, cyclophosphamide, vincristine, and prednisolone) including prednisolone (PSL) sodium succinate 40 mg/body. Ten days after the initiation of R-THP-COP therapy, 75 μg/day of filgrastim (recombinant human G-CSF) was administered for five days for preventing neutropenia. The day after the last filgrastim administration, high fever and right cervical pain developed.

**Figure 1:**
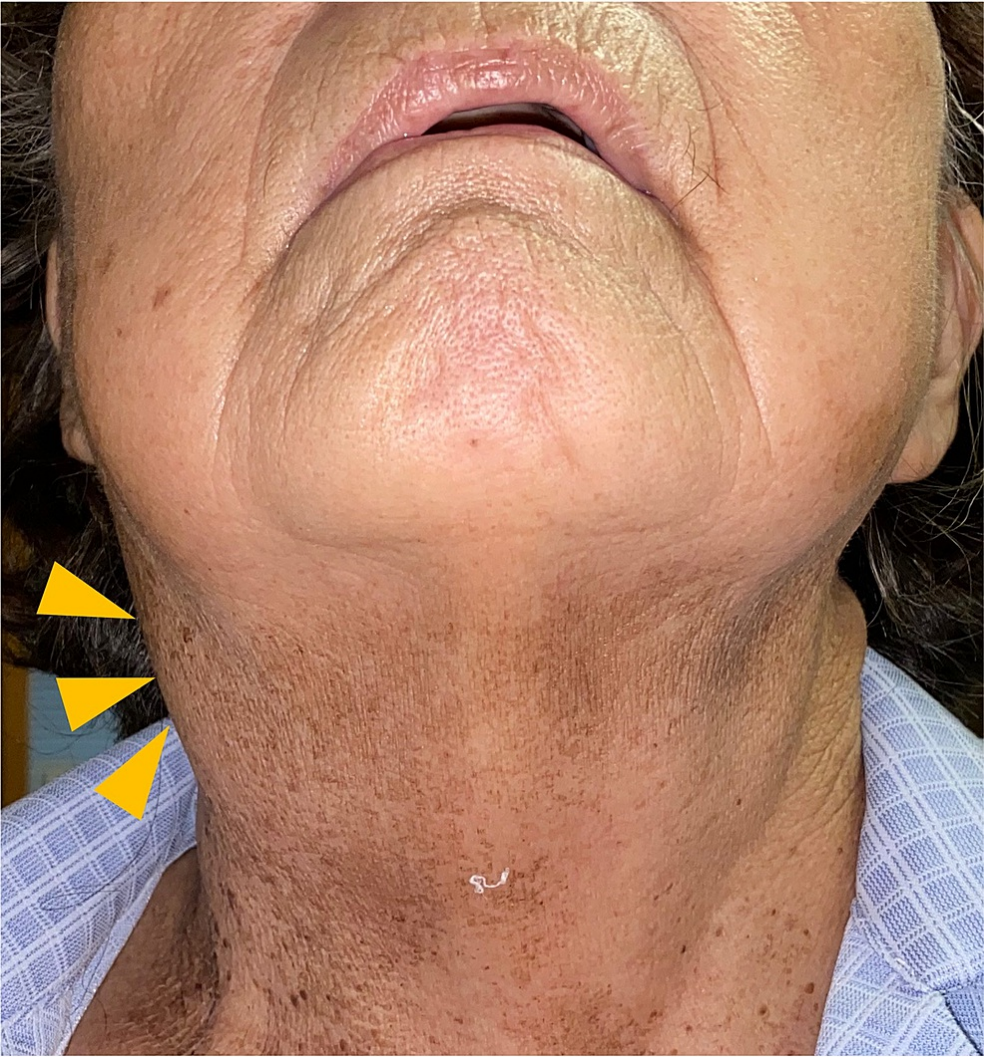
Right cervical swelling was observed, and pain was present in the same area (arrows).

**Figure 2:**
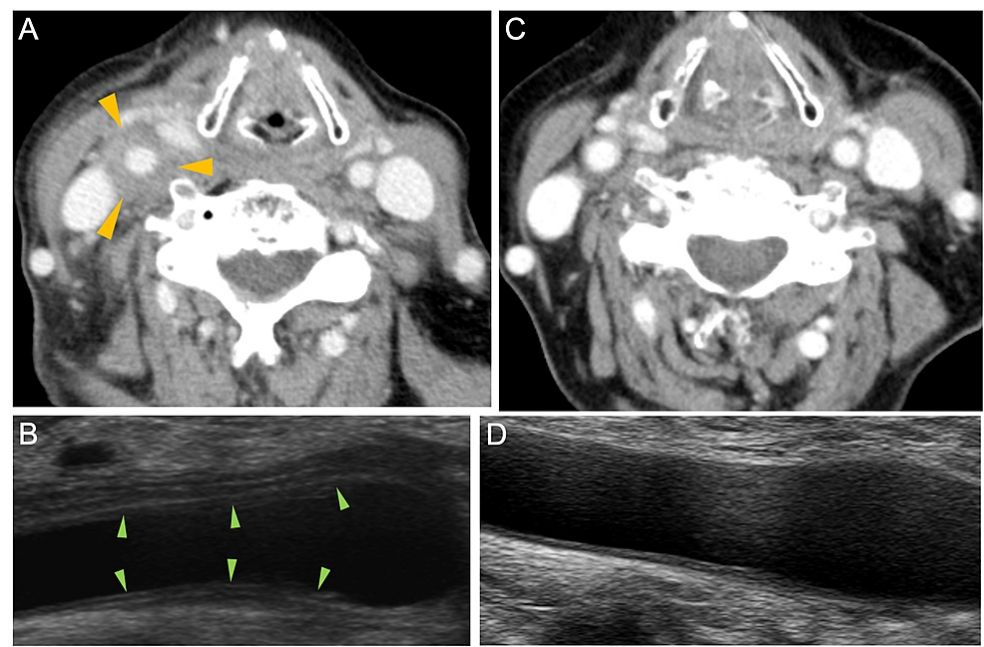
Contrast-enhanced computed tomography and cervical artery ultrasound imaging before (A) and (B) and after (C) and (D) corticosteroid therapy. Contrast-enhanced computed tomography (yellow arrows) and ultrasound imaging (green arrows) revealed wall thickening in the right common carotid artery before treatment. Five weeks after starting treatment, the wall thickening in the right carotid artery had disappeared.

Her vital signs were as follows: body temperature 38.6℃, blood pressure 104/59 mm Hg, heart rate 88 beats/min, and oxygen saturation 99% in room air. Her right cervical region showed severe tenderness and swelling (Figure 1). In the laboratory data on admission (Table 1), stable anemia and thrombocytopenia, hypoalbuminemia, the elevation of serum C-reactive protein (CRP) level and serum complement level, and low level of serum immunoglobulin G were observed; white blood cells were normal; liver function values were slightly elevated; renal function values and serum electrolytes were unremarkable; antinuclear antibody, anti-Sjögren's syndrome A (SSA) antibody, rheumatoid factor (RF), anticyclic citrullinated peptide (CCP) antibody, and antineutrophil cytoplasmic antibody (ANCA) were negative; T-spot, β-D-glucan, and cytomegalovirus (CMV) antigenemia were negative; urinalysis showed no abnormal findings; and blood and urine cultures were negative. Contrast-enhanced computed tomography of the neck, chest, abdomen, and pelvis and cervical artery ultrasound imaging were performed, and wall thickening in the right common carotid artery was detected (Figure 2A-2D).

**Table 1:** Laboratory data on admission. TP: total protein; Alb: albumin; ALT: alanine transaminase; AST: aspartate aminotransferase; ALP: alkaline phosphatase; GTP: glutamyl transferase; LDH: lactate dehydrogenase; BUN: blood urine nitrogen; Cre: creatinine; CRP: C-reactive protein; IgG: immunoglobulin G; IgA: immunoglobulin A; IgM: immunoglobulin M; IgG4: immunoglobulin G4; C3: complement component 3; C4: complement component 4; SSA: Sjögren's syndrome A; RF: rheumatoid factor; CCP: citrullinated peptide; MPO-ANCA: myeloperoxidase-antineutrophil cytoplasmic antibody; PR3-ANCA: proteinase 3-antineutrophil cytoplasmic antibody; CMV: cytomegalovirus.

Hematology		Serology	
White blood cells	6700 /μL	CRP	19.52 mg/dL
Neutrophils	86%	IgG	687 mg/dL
Eosinophils	0.0%	IgA	109 mg/dL
Basophils	0.0%	IgM	37.6 U/mL
Lymphocytes	5%	IgG4	25 mg/dL
Monocytes	8.0%	C3	160.7 mg/dL
Red blood cells	2.95 × 10^6^ /μL	C4	44.6 mg/dL
Hemoglobin	8.0 g/dL	Antinuclear antibody	<40
Hematocrit	25.0%	Anti-SSA antibody	<1.0 U/mL
Platelet	137 × 10^3^ /μL	RF	3.5 IU/mL
		Anti-CCP antibody	0.5 U/mL
Biochemistry		MPO-ANCA	<1.0 IU/mL
TP	5.6 g/dL	PR3-ANCA	<1.0 IU/mL
Alb	2.7 g/dL	T-spot	(−)
ALT	30 U/L	β-D-glucan	(−)
AST	35 U/L	CMV antigenemia	(−)
ALP	154 U/L		
γ-GTP	94 U/L	Urinalysis	
LDH	144 U/L	Protein	(−)
BUN	10 mg/dL	Glucose	(−)
Cre	0.52 mg/dL	Red blood cells	(−)
Na	134 mmol/L	White blood cells	(−)
K	4.2 mmol/L		
Cl	102 mmol/L		
Ca	8.4 mg/dL		

Because she had become a compromised host by chemotherapy, she was initially suspected of the infected aneurysm and treated with cefozopran as empiric therapy. However, her symptoms rapidly deteriorated, and her serum C-reactive protein level elevated to 25.48 mg/dL seven days after fever onset. We diagnosed her with filgrastim-induced vasculitis because there were no findings suggestive of infection or collagen disease, and her symptoms appeared relatively quickly after filgrastim administration. Although the possibility of primary large vessel vasculitis including giant cell arteritis or Takayasu arteritis could not be ruled out, filgrastim-induced vasculitis was considered to be a more appropriate diagnosis because the fever onset was after filgrastim administration.

**Figure 3:**
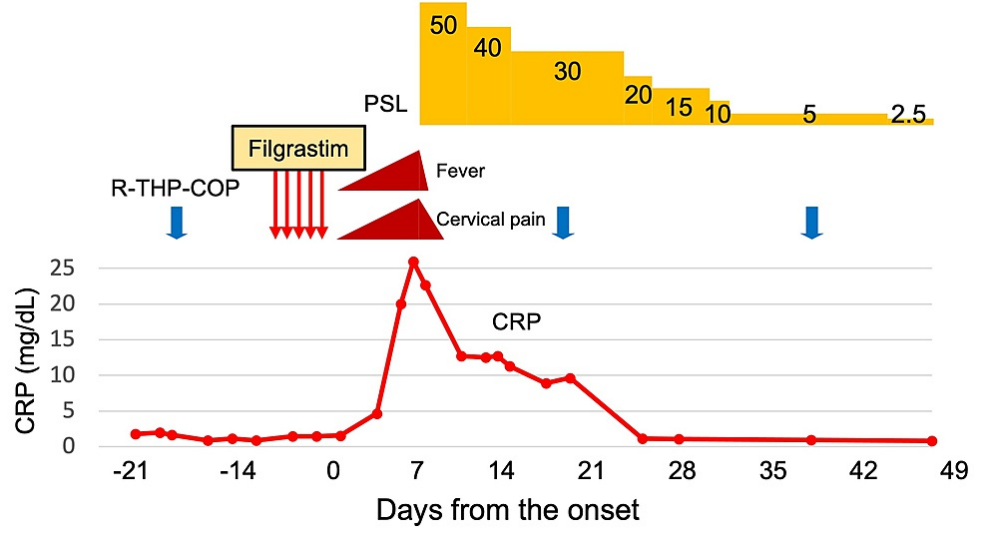
Clinical course. High fever and right cervical pain appeared after five days of filgrastim administration. High-dose corticosteroid therapy remarkably improved the patient’s symptoms and inflammation. R-THP-COP: rituximab, pirarubicin, cyclophosphamide, vincristine, and prednisolone; PSL: prednisolone; CRP: C-reactive protein.

Prednisolone of 50 mg/day (1 mg/kg/day) dramatically improved her symptoms within a few days. One week after starting corticosteroid therapy, the cervical artery ultrasound imaging showed that the wall thickening in the right common carotid artery had greatly improved. After her symptoms disappeared, she was able to receive R-THP-COP therapy without any problems, and the dosage of corticosteroids was promptly reduced. The dosage of prednisolone was successfully reduced to 5 mg/day after five weeks of corticosteroid therapy (Figure 3). The follow-up contrast-enhanced computed tomography and cervical artery ultrasound imaging five weeks after starting corticosteroid therapy revealed an improvement of the vasculitis (Figure 2C, 2D). This rapid response to corticosteroid therapy was different from that for primary large vessel vasculitis. Finally, prednisolone was discontinued six weeks after initiation, and she did not experience vasculitis relapse.

## Discussion

Although G-CSF-induced vasculitis is extremely rare, reports of those in large vessels are abundant. According to an investigation based on a database of Japanese adverse drug events, the incidence of G-CSF-induced vasculitis was 0.47% [3]. Most previous cases have responded to corticosteroids therapy, while the dosage of corticosteroids and the duration of treatment vary across reports, and there is no consistency between them.

G-CSF-induced vasculitis is diagnosed on the basis of the clinical course of disease onset after G-CSF administration. One of the important differential diseases other than G-CSF is drug-induced vasculitis. In particular, chemotherapy-induced vasculitis should be considered because G-CSF is often used in combination with chemotherapy. However, chemotherapy-induced vasculitis is extremely rare, with only a few cases reported with gemcitabine, bevacizumab, or cisplatin [4-6]. In addition, taxanes have been reported to be more frequently administered to patients who developed G-CSF-induced vasculitis [4]. In our case, there were no reports of vasculitis caused by R-THP-COP therapy, and since the vasculitis did not flare up even after continued R-THP-COP therapy, we considered that it had not been caused by chemotherapy.

Recently, Muzzana et al. conducted a systematic review and reported that 47% of G-CSF-induced vasculitis cases were treated with corticosteroids [2]. Although some patients with G-CSF-vasculitis improved with discontinuation of G-CSF alone and without corticosteroid therapy [2,4], aortic dissection and aneurysm have been reported as rare complications, which can appear in an acute course within a few weeks after the onset of vasculitis [7,8]. The cases treated with corticosteroids reached fever resolution within a few days [9-15], while it took one to three weeks for those treated without corticosteroids to reach fever resolution [1,4,13,16-20]. Since G-CSF-induced vasculitis occurs mainly in patients with malignancy, persistent fever due to G-CSF-induced vasculitis leads to the postponement of chemotherapy. Corticosteroid therapy can promote the improvement of vasculitis, which may lead to the prevention of complications. Therefore, we believe that corticosteroid therapy prevents progression to aortic dissection or aneurysm, and allows a safe continuation of chemotherapy.

What is the appropriate initial dose of corticosteroids? In our case, prednisolone was started at 50 mg/day (1 mg/kg/day). Most previous cases were treated with high-dose corticosteroids (≥40 mg/day) [2]. There is a case report of G-CSF-induced vasculitis treated with prednisolone at a dose of 0.5 mg/kg/day, which was later increased to 1 mg/kg/day due to insufficient effects [9]. On the other hand, there is no case report of G-CSF-induced vasculitis with inadequate response to 1 mg/kg/day prednisolone. On the basis of this evidence, starting the treatment of G-CSF-induced vasculitis with high-dose corticosteroids may be appropriate.

The duration of corticosteroid therapy for G-CSF-induced vasculitis varies widely between reports. Several reports have shown that corticosteroid therapy could be completed in a short period of fewer than three months [5,9,14]. Regarding the tapering of corticosteroids, Mukai et al. succeeded in rapidly reducing the dose of prednisolone from 55 mg to 10 mg in three weeks [21]. In our case, we rapidly reduced and discontinued the corticosteroids within six weeks and obtained a complete remission of G-CSF-induced vasculitis without relapse. Since G-CSF-induced vasculitis improves even with discontinuation of G-CSF alone, short-term corticosteroid therapy with rapid dose reduction may be a reasonable choice.

## Conclusions

We have described the case of G-CSF-induced vasculitis successfully treated with short-term corticosteroid therapy. Clinicians should be aware of vasculitis as a possible adverse event of G-CSF and consider starting and rapidly tapering high-dose corticosteroids for G-CSF-induced vasculitis. Further studies are required to clarify the optimal treatment for G-CSF-induced vasculitis.
